# The magnitude of depression in heart failure patients and its association with NYHA class

**DOI:** 10.1192/j.eurpsy.2021.918

**Published:** 2021-08-13

**Authors:** J. Botto, S. Martins, E. Moreira, J. Silva Cardoso, L. Fernandes

**Affiliations:** 1 Fmup, Faculty of Medicine - University Porto, Porto, Portugal; 2 Department Of Clinical Neuroscience And Mental Health And Center for health technology and services research (cintesis), Faculty of Medicine - University Porto, Porto, Portugal; 3 Center For Health Technology And Services Research (cintesis), Faculty of Medicine - University Porto, Porto, Portugal; 4 Department Of Medicine, Faculty of Medicine - University Porto, Porto, Portugal; 5 Department Of Cardiology, Centro Hospitalar Universitário S. João (CHUSJ), Porto, Portugal; 6 Psychiatry Service, Centro Hospitalar Universitário de São João, Porto, Portugal

**Keywords:** NYHA class, heart failure, Depression

## Abstract

**Introduction:**

Depression is commonly present among HF patients and is associated with adverse clinical outcomes. However, research regarding its association with New York Heart Association (NYHA) class is still scarce.

**Objectives:**

To evaluate the presence of depression symptoms in HF outpatients and analyze its association with NYHA class.

**Methods:**

This study is part of a larger research project (Deus Ex-Machina/NORTE-01-0145-FEDER-00026). HF patients were recruited from an outpatient clinic at a University Hospital. Exclusion criteria were: unable to communicate, severe visual acuity deficit or NYHA class IV. Sociodemographic data and NYHA class were registered. The Patient Health Questionnaire-9 (PHQ-9) was used to assess depression, with a score ≥10 indicating clinically relevant depression.

**Results:**

A sample of 136 HF patients was included, with a median age of 59 (range: 24-81) years old, where 66% were men. Almost half of the patients (49%) were in NYHA class II, followed by class I (36%) and class III (15%). The median score of PHQ-9 was 4(range:0-18), with 26% showing clinically relevant depression. PHQ-9 total score was associated with NYHA class (p=0.001), with higher median scores in worse NYHA classes [class I: 3 (IQR: 5.5), class II: 4 (IQR: 8) and class III: 8.5 (IQR:9.3)].

**Conclusions:**

In this study, depression was present in 26% of HF outpatients and was associated with more severe HF symptoms. Consequently, preventing, monitoring, and treating depression in the management of these patients is recommended. Further research is needed for a deeper analysis of this association.

